# Distinct Nasopharyngeal and Oropharyngeal Microbiota of Children with Influenza A Virus Compared with Healthy Children

**DOI:** 10.1155/2018/6362716

**Published:** 2018-11-19

**Authors:** Zhixin Wen, Gan Xie, Qian Zhou, Chuangzhao Qiu, Jing Li, Qian Hu, Wenkui Dai, Dongfang Li, Yuejie Zheng, Feiqiu Wen

**Affiliations:** ^1^Department of Respiratory Diseases, Shenzhen Children's Hospital, No. 7019, Yitian Road, Futian District, Shenzhen 518026, China; ^2^Department of Microbial Research, WeHealthGene Institute, 3C19, No. 19 Building, Dayun Software Town, Shenzhen 518000, China; ^3^Department of Hematology and Oncology, Shenzhen Children's Hospital, Shenzhen 518038, China

## Abstract

**Background:**

Influenza A virus (IAV) has had the highest morbidity globally over the past decade. A growing number of studies indicate that the upper respiratory tract (URT) microbiota plays a key role for respiratory health and that a dysfunctional respiratory microbiota is associated with disease; but the impact of microbiota during influenza is understudied.

**Methods:**

We recruited 180 children, including 121 IAV patients and 59 age-matched healthy children. Nasopharyngeal (NP) and oropharyngeal (OP) swabs were collected to conduct 16S rDNA sequencing and compare microbiota structures in different individuals.

**Results:**

Both NP and OP microbiota in IAV patients differed from those in healthy individuals. The NP dominated genera in IVA patients, such as* Moraxella*,* Staphylococcus*,* Corynebacterium*, and* Dolosigranulum*, showed lower abundance than in healthy children. The* Streptococcus *significantly enriched in patients' NP and* Phyllobacterium* could be generally detected in patients' NP microbiota. The most abundant genera in OP microbiota showed a decline tendency in patients, including* Streptococcus, Neisseria*, and* Haemophilus.* The URT's bacterial concurrence network changed dramatically in patients. NP and OP samples were clustered into subgroups by different dominant genera; and NP and OP microbiota provided the precise indicators to distinguish IAV patients from healthy children.

**Conclusion:**

This is the first respiratory microbiome analysis on pediatric IAV infection which reveals distinct NP and OP microbiota in influenza patients. It provides a new insight into IAV research from the microecology aspect and promotes the understanding of IAV pathogenesis.

## 1. Introduction

Various respiratory viral agents, such as influenza A virus (IAV), adenovirus (ADV), and respiratory syncytial virus (RSV), are common pathogens which cause childhood community-acquired pneumonia (CAP) [1]. In the past ten years, influenza has caused a high morbidity ratio in China and other countries globally, especially in 2017-2018 [2, 3]. Several studies reported on the mechanisms of IAV, such as virulence [4], evolution [5], and host-virus interaction [6]. However, only a few studies reported microbiome changes during influenza infection and these were mainly conducted in adult or aged populations [7–10].

The upper respiratory tract (URT) functions as an interface between exterior environments, lung and gastrointestinal tract [11], and primes the host immune system to protect the mucosal surface from pathogenic infection [12, 13]. Several reviews summarized the viral-bacterial superinfection pathway [14, 15], which indicate that microbiota dysbiosis is associated with pathogen invasion, escaping the host immune system and finally leading to respiratory disease.

Recently, several reports have indicated that healthy and diseased children have different nasopharyngeal (NP) and oropharyngeal (OP) microbiota [16–18]. Moreover, this distinct respiratory microbiota pattern varied with infectious pathogens [19]. For instance, the health-associated commensals* Corynebacterium* and* Dolosigranulum* are significantly decreased in* Mycoplasma pneumoniae *pneumonia patients [18]. Dominant* Haemophilus*/*Streptococcus* in NP microbiota indicate a high risk of respiratory infection [20] or more severe bronchiolitis [21]. RSV patients' microbiota in the respiratory tract also revealed a significant alteration [22], with dominant* Streptococcus pneumonia* and* Staphylococcus aureus* associated with historical IAV pandemics [23]. Furthermore, Wouter* et al*. demonstrated that the abundant lactobacilli,* Rothia* or* S. pneumoniae,* were positively related to pneumonia severity index (PSI) [16].

These findings suggest the importance of the bacterial community during respiratory viral infection. However, how the bacterial community of the URT changes in pediatric IAV remains to be explored. In this study, we investigated NP and OP microbiota using 16S-based sequencing and aimed to identify alterations in IAV patients compared to healthy children. Bacterial markers to distinguish IAV patients and healthy children were also explored.

## 2. Materials and Methods

### 2.1. Ethical Approval

This study was approved by the Ethical Committee of Shenzhen Children's Hospital (registration number 2016013). Guardians of recruited children provided informed consent. All procedures performed in this study were in accordance with ethical standards of the institutional and/or national research committee, as well as the 1964 Helsinki declaration and its subsequent amendments, or comparable ethical standards.

### 2.2. Subjects Inclusion and Pathogen Detection

All children with suspected IAV infection were enrolled at the fever outpatient clinic at Shenzhen Children's Hospital. Patients with confirmed IAV who met the following criteria were selected: (i) typical clinical symptoms of acute influenza (fever, cough, dyspnea, and vomiting); (ii) positive detection of IAV by rapid PCR-fluorescence probing (DaAnGene, Guangzhou, China); (iii) no antibiotic administration before fever clinic; (iv) severe cases, defined by severity of symptoms and those who were admitted to the intensive care unit (ICU).

NP microbial samples were collected by skilled clinicians during the outpatient evaluation with specific swabs (25-800-A-50, Puritan, Guilford, North Carolina, USA). At the same time, OP microbial samples were also collected by specific swabs (155C, COPAN, Murrieta, USA) [24]. Common pathogens were detected by the following methods: bacterial culturing was conducted to detect clinical common bacterial pathogens [25] and nucleic acid testing (NAT) was applied to identify viral or atypical pathogens as described previously [18]. Unused swabs and DNA extraction kits were utilized as negative controls to assess for experimental contamination [18]. All specimens were frozen at –80°C for microbial sequencing.

Healthy children were recruited after physical examination at Shenzhen Children's Hospital [24], according to the following criteria: no asthma or family history of allergy; no history of pneumonia; no wheezing, fever, cough, or other respiratory/allergic symptoms; no antibiotic exposure for 1 month prior to this study; and no disease symptoms within 1 week of sampling.

### 2.3. DNA Extraction, PCR, and Sequencing

Microbial genomic DNA was extracted using the Power Soil DNA Isolation Kit (Mo Bio Laboratories, Carlsbad, CA, USA). PCR amplicon libraries were constructed by the V3-V4 hypervariable region of the 16S rDNA gene [26]. Qualified libraries were then sequenced by the Illumina MiSeq sequencing platform (Illumina, San Diego, CA, USA) [27].

### 2.4. Bioinformatics Analysis and Visualization

Raw sequencing data were processed by the QIIME pipeline [28]. Data filtration, representative taxa assignment, and diversity calculations were conducted according to our previous study [29]. A rarefaction curve of OTU was used to assess the saturation of sequencing data. Principal component analysis (PCA) was used to distinguish IAV samples from healthy ones. The statistical difference of characters between patients and healthy children was using chi-square test (*p* value). The comparison of bacterial relative abundance between patients and healthy children was performed by Wilcoxon rank-sum test and the significance of multiple comparisons was adjusted by FDR (*q*-value). Distances between samples were calculated by Bray-Curtis dissimilarity and then clustered by the hierarchical method. The visualization of cluster topology used iTol [30], and the best subgroup was divided by the Silhouette method. The community concurrence network was constructed based on the correlation of microbiota by Spearman's rank correlation coefficient [18]. The prediction model was selected by the random forest method and cross-validation was used to select biomarkers for predicting disease versus health [31]. Receiver-operating characteristic (ROC) curves and the area under the curve (AUC) for each crucial genus were applied to assess the accuracy of biomarkers [9]. All other data visualization was produced by the package** “**ggplot2**”** of R software (v. 3.2.3) and Cytoscape (v. 3.4.0).

## 3. Results

### 3.1. Sample Information, Data Output, and Confounder Analysis

We enrolled 180 children, including 121 patients with IAV and 59 age-matched healthy children. Patients with IAV had no history of allergy, pneumonia, or asthma. Characteristics of the study population are summarized in Table 1. Based on disease severity, 11 patients were classified as severe and admitted to the pediatric ICU (Table S1). The confounder analysis indicated that IAV infection is the most significant factor to explain variations in microbial samples.

A total of 9,018,174 high-quality tags were produced, averaging 25,592 (6,911–29,192), 26,596 (17,368–29,218), 26,063 (18,303–29,272), and 21,493 (9,724–28,738) for the NP-IAV (NP-P), OP-IAV (OP-P), NP-health (NP-H), and OP-health (OP-H) groups, respectively. The average OTU numbers in the NP-P, OP-P, NP-H, and OP-H groups were 1,004, 383, 255, and 143, respectively (Supplementary Figure 1).

### 3.2. NP and OP Microbiota of Patients Differ Significantly from Those of Healthy Children

**Figure 1:**
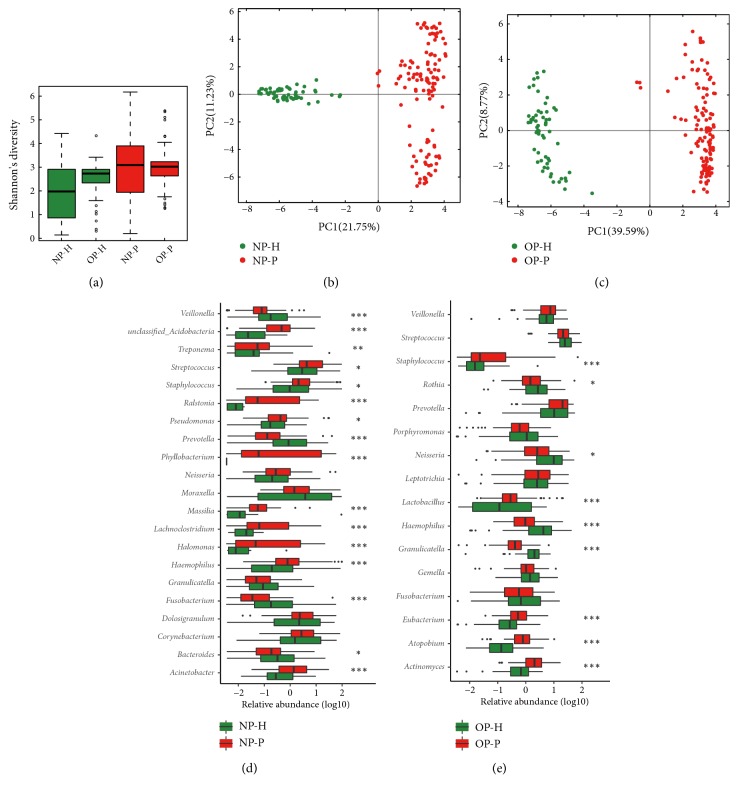
NP/OP microbiota structure in IAV patients and healthy children. (a) Shannon index of NP and OP microbiota in patients and healthy children. (b, c) Principal components analysis (PCA) of NP/OP samples. (d, e) Comparison of dominated genera of NP/OP microbiota between patients with IAV and healthy children. The vertical axis represents genus name, and the horizontal axis shows the log⁡10 value of relative abundance. *∗*, *∗∗*, and *∗∗∗* represent* q*-values ⩽ 0.05, ⩽ 0.01, and ⩽ 0.001, respectively. Objects painted green or red represent healthy or disease samples.

The diversity of the microbioal community in the NP-P/OP-P group was significantly higher than that of the NP-H/OP-H group (p value <0.001) (Figure 1(a)). The diversity of the OP microbiota was also higher than that of the NP microbiota, in both healthy and diseased children (Figure 1(a)). Microbial samples in patients with IAV were clearly separated in clustering compared to healthy controls (Figures 1(b) and 1(c)).

Firmicutes predominates in both the NP and OP microbiota of healthy or diseased children (NP: 39.17% in IAV patients vs. 43.14% in healthy children,* q*-value=0.594; OP: 47.27% in IAV patients vs. 46.50% in healthy children,* q*-value=0.730) (Table S2). Proteobacteria accounts for a secondary proportion of NP microbiota in the disease group. On the contrary, the abundance of Proteobacteria was significantly lower in the OP-P group (*q*-value=0.030). Actinobacteria significantly changed in both NP (*q*-value=0.023) and OP (*q*-value =0.001) microbiota during IAV infection (Table S2).

At the genus level, the abundant* Moraxella* of NP exhibited a decline tendency in IAV patients compared to healthy children, followed by* Staphylococcus* (*q*-value=0.018),* Corynebacterium*, and* Dolosigranulum* (Figure 1(d), Table S3). On the other hand,* Streptococcus* was enriched significantly in NP-P (16.79% vs. 10.09% in NP-H,* q*-value=0.028) while* Phyllobacterium* could only be detected in patients with IAV (6.94% vs. 0.00% in NP-H,* q*-value <0.001) (Figure 1(d), Table S3). Other dominant genera such as* Acinetobacter*, unclassified* Acidobacteria*,* Ralstonia*,* Pseudomonas*,* Lachnoclostridium*, and* Halomonas* increased signficantly in the IAV group (Figure 1(d), Table S3).

In OP microbiota, seven of the top 10 genera showed lower abundance in patients, including* Streptococcus*,* Neisseria* (13.06% vs. 6.05%,* q*-value=0.017),* Haemophilus* (6.29% vs. 1.59%,* q*-value<0.001),* Rothia* (4.34% vs. 3.08%,* q*-value=0.045),* Fusobacterium*,* Granulicatella* (2.11% vs. 0.68%,* q*-value<0.001), and* Gemella*. The remaining three genera,* Prevotella*,* Veillonella*, and* Leptotrichia*, were amassed during IAV infection (Figure 1(e), Table S3).* Lactobacillus*,* Eubacterium*,* Atopobium*, and* Actinomyces* show a significant increment in ED (Figure 1(e), Table S3).

### 3.3. NP and OP Concurrence Bacterial Network Altered in Patients

**Figure 2:**
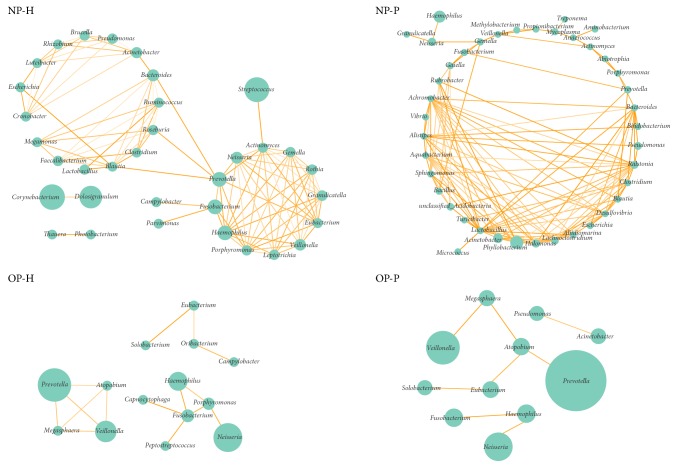
Co-occurrence network of NP/OP microbiota in patients with IAV and healthy ones. The circle size represents relative abundance, and the density of the dashed line represents the Spearman coefficient.

In NP-H, the concurrence bacterial network (Figure 2) consisted of 34 genera and could be grouped into two subnetworks with three hub nodes, namely, Prevotella, Roseburia, and Bacteroides. However, the NP microbial interaction shaped to a more complex network with more but different genera (44 genera; 16 genera also represented in NP-H). The structure of the microbial interaction in the OP tended to be simpler and linear during influenza onset (Figure 2).

### 3.4. Different Composition of Microbiota in Subclusters of NP and OP, but No Specific Pattern Related to Severe Case

**Figure 3:**
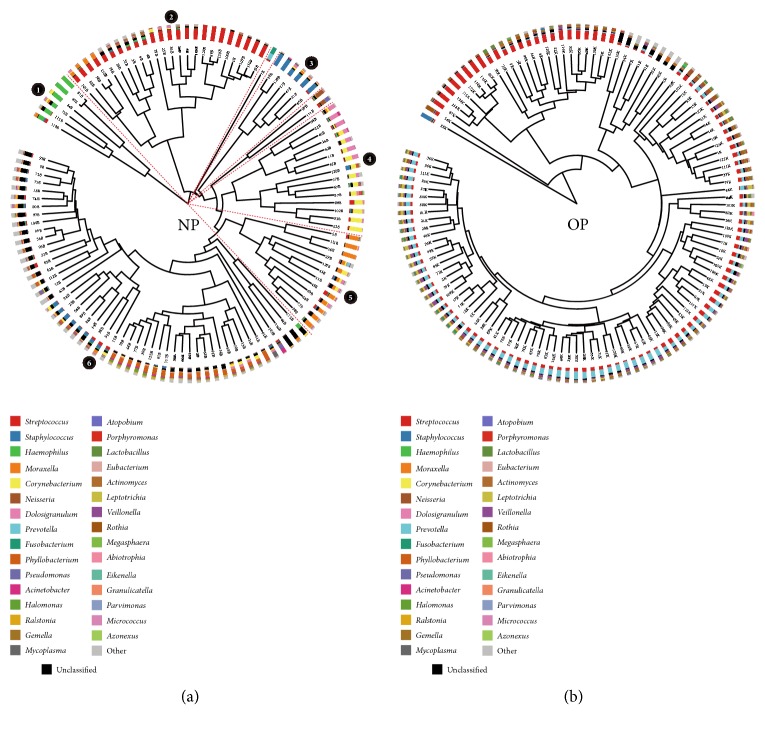
Hierarchical clustering analysis of microbiota in the NP and OP. The circle dendrograms were constructed based on the dissimilarity of microbiota composition between samples. Adjacent to the dendrogram branch ends, stacked bar charts show the relative abundance of the dominant genera in the NP and OP. Subclusters (defined as more than three samples) are designated by the dotted red lines originating from the center of the dendrogram.

With hierarchical clustering, NP samples could be divided into 6 subsets (G1 to G6) (Figure 3(a)), except that the two subbranches consist of only one or two samples. The dominanted genera in each subset are* Haemophilus* (66.05%, G1 contains 6 samples),* Streptococcus* (63.77%, G2 contains 24 samples),* Staphylococcus* (65.80%, G3 contains 6 samples), mixed* Corynebacterium-Dolosigranulum* (39.22% and 24.95%, G4 contains 15 samples),* Moraxella* (45.16%, G5 contains 12 samples), and mixed* Zoogloea-Phyllobacterium* (24.93% and 13.85%, G6 includes 51 samples). Although OP samples could not be clearly separated into subclusters, several patterns of specific bacterial components were recognized, such as* Streptococcus*-dominant,* Prevotella*-dominant, and mixed genera with several dominating (Figure 3(b)). The 11 severe cases admitted to the ICU included P1, P3, and P32 assigned to G2, P53 to G4, P111 in the small branch (with only two samples), and the other six patients (P2, P23, P56, P88, P103, and P110) to G6. There was no correlation between disease severity and bacterial composition, and no relevant genus related to severe illness.

### 3.5. Key Genera Served as Biomarkers to Provide Prediction Model for IAV Infection

**Figure 4:**
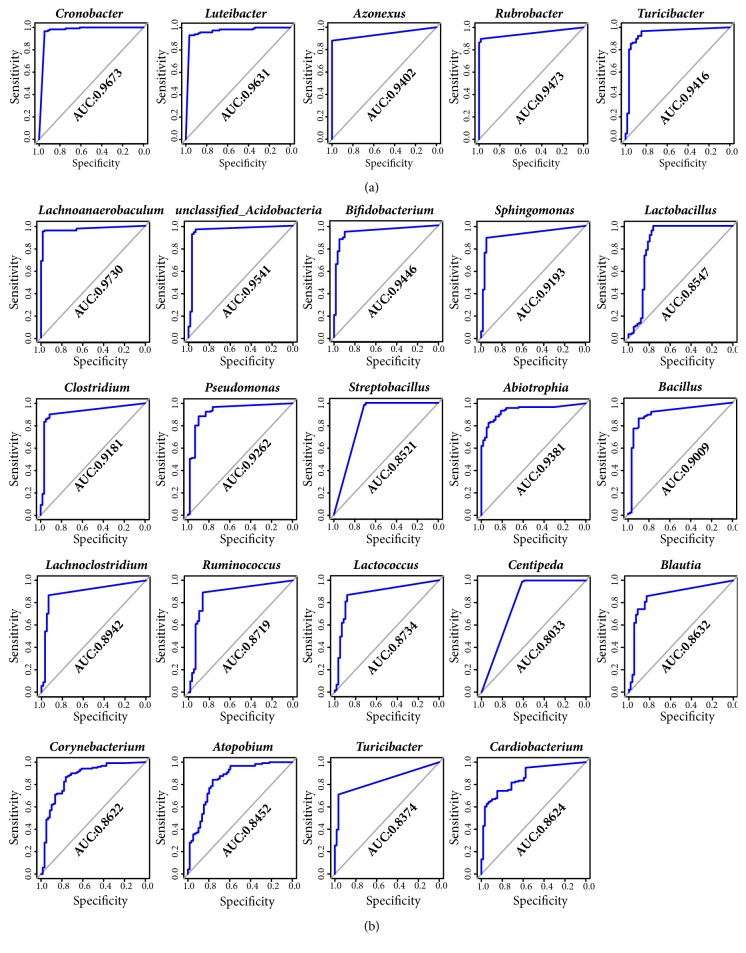
Prediction of biomarkers in the NP/OP microbiome. Receiver-operating characteristic (ROC) plots were used to estimate the efficiency of five key genera in NP (a) and 19 key genera in OP (b). The area under curve (AUC) of each genus shows the high accuracy to distinguish patients with IAV from healthy controls.

Key genera were identified by random forest analysis to distinguish patients with IAV from healthy controls based on URT microbiota composition. After cross-validation, five NP genera, including* Cronobacter*,* Luteibacter*,* Azonexus*,* Rubrobacter*, and* Turicibacter*, were found to be the most important indicators to discriminate between patients with IAV and healthy controls, with greater predictive efficiency (AUC values of 0.967, 0.963, 0.940, 0.947, and 0.942, respectively) (Figure 4(a)). In the OP microbiota, 19 genera were identified as biomarkers, and nine of those showed high accuracy (AUC* >*0.900) (Figure 4(b)), especially for* Lachnoanaerobaculum* (AUC =0.973) (Figure 4(b)).

## 4. Discussion

The respiratory tract is colonized by various microbes [32], plays an important immune function during childhood, and is a defender against pathogen invasion [33, 34]. During respiratory infection, the microecology becomes unstable, resulting in dysbiosis of the virus-bacteria community [14]. NP microbial commensals primarily extract barren nutrients from the respiratory epithelium [35] and are easily influenced by the exterior environment [36]. The diversity of NP microbiota significantly increased in patients with IAV compared to healthy children, but decreased in bacterial respiratory infection [37, 38]. This may be due to virus-specific characters, such as suppressing bacterial clearance, inflow of nutrients, and promotion of bacterial outgrowth [15]. Contrary to the NP, the OP microbial environment is abundant with nutrition due to food ingestion and esophageal reflux, which implicates a more stable and massive microbiota than that of the NP niche [39, 40]. Therefore, the diversity of the OP microbiota shows a nonobvious changing tendency in patients with IAV. This can be partly explained by specific clearance mechanisms for different pathogens in different respiratory niches [41].

The predominant* Corynebacterium* and* Dolosigranulum* in the NP microbiota were strongly associated with a reduced ratio of acute otitis media [42], which have shown decline tendency in IAV patients.* Moraxella*, which is associated with lower risk of respiratory infection [34], also occupied lower abundance in patients than in healthy children. On the contrary,* Streptococcus* has been shown to be the most abundant, by synergistic stimulation of type I interferons during influenza virus infection [43] and resulting in pneumonia susceptibility [32]. The suddenly enriched* Phyllobacterium*, which could not be detected in healthy NP samples, have not been reported in human diseases until now. The OP bacterial community also changed dramatically and is inconsistent with a previous adult IAV microbiota study [9], in which* Bacteroidetes* is the most abundant, whether in healthy or diseased patients, followed by* Proteobacteria* and* Firmicutes*. It is implicated to have a different composition in children and adults during the aging process [44]. As a result, the core bacterial concurrence network was reconstructed unrecognizably in the NP and is simpler in the OP of IAV children.

According to recent studies, the microbiota can be further divided into subgroups. Alexande et al. indicated that patients with IAV represent individual specific microbiota compositions [10]. Adults or elderly patients can be clustered into several subgroups, whose URT microbiota dominated by* Streptococcus* was strongly associated with the pneumonia severity index [16]. In our study, NP microbiota was also separated into subgroups with group-specific dominated bacteria, but no specific pattern related to the severe cases.

Luna et al. indicated that the nasal microbiota could be used to detect disease severity [45]. In this study, we also found microbial indictors to precisely distingush those with IAV and healthy children. Until now, neuraminidase inhibitors, such as the well-known oseltamivir, were recommended to treat and prevent influenza, although with an increased risk of side effects [46]. Although the vaccination is currently the best prevention method, we also noticed that mispredicted pandemic influenza strains over the past year led to a worldwide medical burden. On the other hand, the rapid evolution of influenza could disable the preexisting vaccination [47]. Combining valuable biomarkers with the microbial characteristics of other pediatric URTs will provide more comprehensive information for influenza prevention and prediction.

There were several limitations to this study. No precise types of IAV strains were reported due to the clinical practice of using a generic influzena A virus detection kit; this might have partly impacted the NP subgroups' microbiota composition. Also, all samples were collected when patients first presented to the fever clinic; we could not determine patterns existing between severe cases and mild cases. Though we identified predictive indicators to distinguish IAV and healthy individuals, it might be difficult to identify IAV among other respiratory virus infections. Lastly, 16S rDNA analysis cannot identify species-level pathogens, nor explain functional dysbiosis in the infection-caused microbiota disorder.

## 5. Conclusions

This study is the first research on the microbiota of pediatric patients with IAV infection and is thus an important reference for understanding the respiratory microbiota in this population. These findings reveal the dysbiosis of the microbiota of URTs in children with IAV and provide new insight into the pathogenesis of IAV.

## Figures and Tables

**Table 1:** Sample information.

	Healthy Children	IAV Patients	
	(n = 59)	(n = 121)	
*Characteristics*			*P-value*
Gender			
Female	33(55.9%)	47(38.8%)	0.045
Male	26(44.1%)	74(61.2%)	
Age (years)^*∗*^	2.8(0.1–9.9)	2.9(0.1–13.8)	
Delivery Mode			
Caesarean section	20(33.9%)	39(32.2%)	0.365
Vaginally born	39(66.1%)	82(67.8%)	
Feed Pattern			
Breast feed	18(30.5%)	68(56.2%)	0.004
Breast feed + Milk feed	31(52.5%)	42(34.7%)	
Milk feed	10(16.9%)	11(9.1%)	
Family history of allergy	-	-	
History of pneumonia	-	-	
Asthma	-	-	
*Clinical records*			
Fever	-	116(95.9%)	
Fever duration(days)^*∗*^	-	2(1-12)	
Cough	-	83(68.6%)	
Cough duration(days)^*∗*^	-	2(1-30)	
WBC (5-12%)	NA	80(66.1%)	
hsCRP (⩽0.5 mg/l)	NA	26(21.5%)	
PCT (<0.5 ng/ml)	NA	61(50.4%)	

“NA” represents not available; CRP, C-reactive protein; PCT, Procalcitonin; “-” represents not detected; “*∗*”: this feature is described with median (range).

## Data Availability

The 16S sequencing data used to support the findings of this study have been deposited in the GenBank repository under accession numbers SRP090593 (healthy children) and SRP149307 (children with IAV).

## Supplementary Materials

Supp lementary Material 1 Table S1: sample information of patients with influenza A virus and healthy children. Table S2: Wilcoxon rank-sum test results of the NP and OP at the phylum level between patients with IAV and healthy children. Table S3: Wilcoxon rank-sum test result of the NP and OP at the genus level between patients with IAV and healthy children. Table S4: detailed genus profile of all samples.

Supplementary Material 2 Figure S1: the rarefaction of samples shows the sequencing data is saturated.
