# Supplementary figures and images for: Distinct Nasopharyngeal and Oropharyngeal Microbiota of Children with Influenza A Virus Compared with Healthy Children
Source: Biomed Res Int. 2018 Nov 19;2018:6362716. doi: 10.1155/2018/6362716 (PMC6276510; doi:10.1155/2018/6362716)

# The Rarefaction of Samples

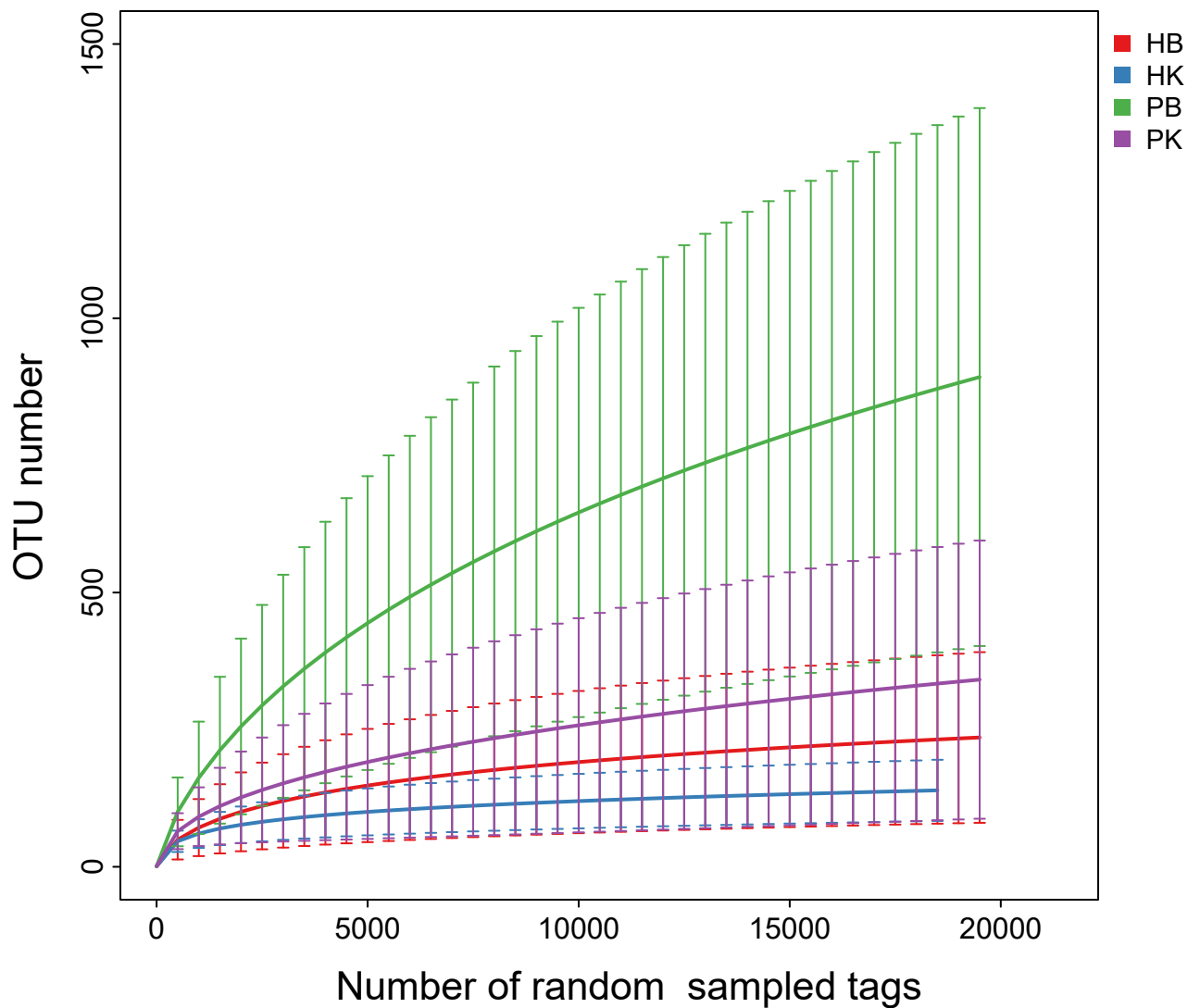

Supplement: Supp lementary Material 1 — Table S1: sample information of patients with influenza A virus and healthy children. Table S2: Wilcoxon rank-sum test results of the NP and OP at the phylum level between patients with IAV and healthy children. Table S3: Wilcoxon rank-sum test result of the NP and OP at the genus level between patients with IAV and healthy children. Table S4: detailed genus profile of all samples. [file 6362716.f1.pdf]
